# Dissecting the Anticancer Mechanism of Trifluoperazine on Pancreatic Ductal Adenocarcinoma

**DOI:** 10.3390/cancers11121869

**Published:** 2019-11-26

**Authors:** Can Huang, Wenjun Lan, Nicolas Fraunhoffer, Analía Meilerman, Juan Iovanna, Patricia Santofimia-Castaño

**Affiliations:** Centre de Recherche en Cancérologie de Marseille (CRCM), INSERM U1068, CNRS UMR 7258, Aix-Marseille Université and Institut Paoli-Calmettes, Parc Scientifique et Technologique de Luminy, 163 Avenue de Luminy, 13288 Marseille, France; can.huang@inserm.fr (C.H.); wenjun.lan@inserm.fr (W.L.); nicolas.fraunhoffer@inserm.fr (N.F.); meilerman.analia@gmail.com (A.M.); juan.iovanna@inserm.fr (J.I.)

**Keywords:** trifluoperazine, cell stress, proteasome inhibitor, drug combination, pancreatic ductal adenocarcinoma (PDAC), repurposing

## Abstract

Pancreatic ductal adenocarcinoma (PDAC) is one of the most aggressive cancers with almost no curative chemotherapeutic treatment. Besides the development of new compounds, repurposing of approved drugs to treat cancer, alone or in combination, has become an attractive strategy, showing many therapeutic and economic advantages. However, it is necessary to improve our knowledge about the mechanism of cell death elicited by approved drugs itself, but also to rationally develop more powerful multidrug treatments. In this work, we focus our attention on determining the mechanism promoting cell death following trifluoperazine (TFP) treatment, which is an antipsychotic drug with strong anticancer activity in PDAC. We demonstrate that TFP induces cell death by apoptosis and necroptosis, which can be partially inhibited by Z-VAD-FMK as well as necrostatin-1, respectively. This cell death promotion is triggered by a poor ATP content, observed in TFP-treated cells as a consequence of a dramatic decrease in OXPHOS metabolism due to mitochondrial stress. Remarkably, mitochondrial homeostasis was seriously affected, and a loss of mitochondrial membrane potential and ROS overproduction was observed. Moreover, this mitochondrial stress was coupled with an ER stress and the activation of the endoplasmic-reticulum-associated protein degradation (ERAD) and the unf olded protein response (UPR) pathways. We took advantage of this information and inhibited this process by using the proteasome inhibitors MG-132 or bortezomib compounds in combination with TFP and found a significant improvement of the anticancer effect of the TFP on primary PDAC-derived cells. In conclusion, this study not only uncovers the molecular mechanisms that are triggered upon TFP-treatment but also its possible combination with bortezomib for the future development of therapies for pancreatic cancer.

## 1. Introduction

Pancreatic adenocarcinoma (PDAC) is one of the most lethal cancers, with a poor prognosis and low life expectancy. Concerning the available treatments, surgery is the only potentially curative treatment, but only 15–20% of patients can be resected, and chemotherapy offers limited benefits [1]. Therefore, present and future research should be focused on improving PDAC therapies to find effective treatments. In the last decades, the repurposing of approved drugs to treat cancer has become an attractive strategy. This approach uses compounds with a known risk, which allows faster access of patients to drugs and lower development costs. This was the case of drugs like celecoxib, metformin or sulindac, which went through clinical trials [2]. Nevertheless, many other compounds can be repositioned in the near future for treating cancer, alone or in combination with other chemotherapy agents. In this regard, trifluoperazine (TFP) is a well-known member of the phenothiazine class of antipsychotic drugs. TFP has shown in vivo anticancer activity in glioblastoma [3], colorectal cancer [4], and triple-negative breast cancer [5], among others. Subsequently, several studies have demonstrated that TFP can inhibit cell migration and proliferation, promote cell cycle arrest, induce apoptosis, and inhibit autophagy in multiple cancer cell lines [6,7]. Recently, we found that TFP binds and mimics the effect of the genetic inhibition of NUPR1, a stress protein that promotes tumor growth and acts as a strong anticancer drug in vitro and in vivo in xenografted nude mice [8]. Therefore, the effect of TFP against NUPR1 can explain, at least in part, its anticancer effect.

Nowadays, the general strategy is to treat patients with cancer with a combination of anti-cancer drugs. Combinational therapy has different purposes, (i) to improve the therapeutic response, attempting to overcome the tumor heterogeneity; (ii) to decrease the doses, and the derived side-effect of each drug; and (iii) to reduce tumor resistance. TFP, as an antipsychotic agent, has some undesirable neuroleptic side-effects as a sole treatment, but lower doses could improve the effectiveness of the anticancer drug and reduce the side-effects. TFP has shown promising anticancer effects in combination with other drugs in vivo, for example, in combination with gefitinib in CL97 cells xenografted mice [9] or increasing radiosensitivity in primary glioblastoma cells in orthotopic xenograft models by impairing homologous recombination [10]. In this line, we focus our attention on determining the mechanisms of action of the repositioned drug TFP, which induced cell death in PDAC, to rationally propose a possible combination of TFP with drugs used in clinical settings. In this work, we identify that TFP induces cell death by apoptosis and necroptosis due to a coupled endoplasmatic reticulum and mitochondrial stress. More importantly, protein degradation by the ubiquitin-proteasome system (UPS) may behave as a salvage pathway for these phenomena. This finding led us to efficiently test the use of proteasome inhibitors in combination with TFP. Thus, our work discloses the molecular mechanisms that induces tumoral cell death upon TFP-treatment and also its possible clinical use in combination with bortezomib, both FDA-approved drugs. Repurposing them as combinatory therapy may lead to developing new treatments for pancreatic adenocarcinoma.

## 2. Results

### 2.1. Trifluoperazine Induces Both Necrosis and Apoptosis in Pancreatic Cancer Cells

**Figure 1:**
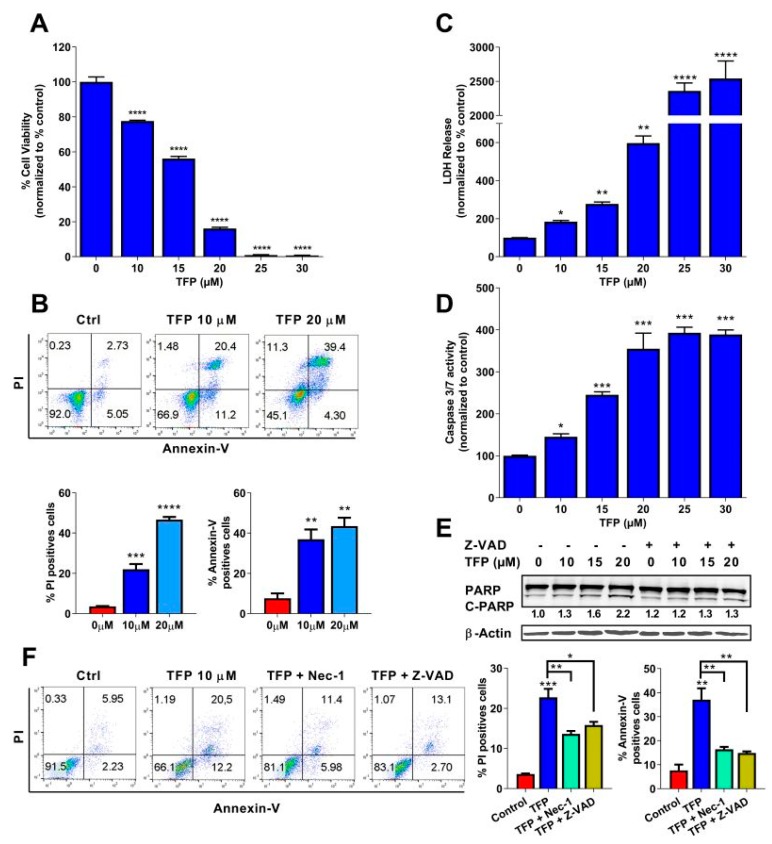
Trifluoperazine (TFP) induced cell death via apoptosis and necroptosis in MiaPaCa-2 cells. (**A**) Viability of MiaPaCa-2 cells upon a 24 h treatment with TFP at increasing concentrations of Trifluoperazine. (**B**) Flow cytometry experiments of annexin V and PI staining upon 24 h of treatment with 10 or 20 μM of TFP, a representative experiment of the dot plot profile of cells is shown. The percentage of PI and annexin V-positive cells is shown. (**C**) LDH release and (**D**) caspase 3/7 activity at increasing concentrations of TFP were measured. (**E**))Western blotting was performed against PARP-1 (upper panel) and β-actin (lower panel) after 24 h of treatment at increasing concentrations of TFP in the presence or not of Z-VAD-FMK (20 μM). (**F**) Flow cytometry was performed on MiaPaCa-2 cell line with 10 μM TFP and in the presence or absence of Z-VAD-FMK (20 μM) or/and necrostatin-1 (Nec-1, 40 μM) during 24 h. Percentages of annexin V-positive cells and PI-positive cells are shown. Statistical significance: * *p* < 0.05, ** *p* < 0.01, *** *p* < 0.001, and **** *p* < 0.001 compared with untreated cells (1-way ANOVA, Tukey’s post hoc test). Data represent mean ± SEM, *n* = 3 (with technical triplicates).

In order to assess the antitumoral activity of TFP in PDAC, we performed a cell viability assay in 96-well plates and treated MiaPaCa-2 cells for 24 h. The results showed that TFP induced cell death in a dose-dependent manner (Figure 1A). Moreover, cell viability was measured upon treatment in 11 PDX-derived cell lines. All the cancer cell lines tested showed a strong sensibility upon treatment, with IC_50_ ranging from 7.59–15.75 µM (Figure S1). We then used flow cytometry after co-labelling cells with propidium iodide (PI) and annexin V treated with TFP to measure necrotic and/or apoptotic events. Treatment with growing concentrations of TFP induced an increase of PI-positive cells (from 3.62% ± 0.15% in non-treated cells to 22.08% ± 2.60% or 46.63% ± 1.33% at 10 or 20 µM of TFP, respectively) as well and annexin V positive cells (from 7.62% ± 2.46% in non-treated cells to 37.06% ± 4.72% or 43.43% ± 4.20% at 10 or 20 µM of TFP, respectively), as shown in Figure 1B. Moreover, to confirm this pro-necrotic and pro-apoptotic effect, Lactate dehydrogenase (LDH) release and caspase 3/7 activity were measured. LDH release and caspase 3/7 activity were strongly increased in TFP-treated cells (Figure 1C,D). In addition, PARP-1 cleavage fraction, which thought to be a marker of apoptosis, also showed an increasing trend with a higher concentration of TFP treatment and rescued by the pan-caspases inhibitor Z-VAD-FMK (Figure 1E). Interestingly, pretreatment with Z-VAD-FMK or the necroptosis inhibitor (Necrostatin-1) were able to rescue MiaPaCa-2 cells and therefore, reduced the percentage of PI-positive cells (from 22.08% ± 2.60% in 10 µM TFP-treated cells to 13.62% ± 0.72% when co-treated with necrostatin-1 or 15.84% ± 0.81% when co-treated with Z-VAD) and annexin V positive cells (from 37.06% ± 4.72% in 10 µM TFP-treated cells to 16.41% ± 1.03% in the presence of necrostatin-1 or 14.88% ± 0.66 when Z-VAD was added) (Figure 1F). Altogether, these data indicate that treatment with TFP induces apoptosis and necrosis of MiaPaCa-2 cells.

### 2.2. Trifluoperazine Decreases the Intracellular ATP Production

**Figure 2:**
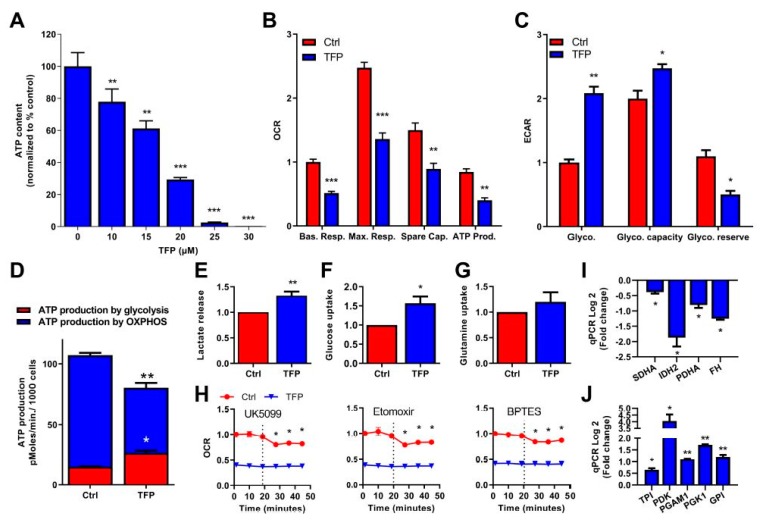
Trifluoperazine decreased ATP production in MiaPaCa-2 cells. (**A**) Cells were incubated with TFP at increasing concentrations and ATP production was measured after 24 h of treatment. (**B**) OXPHOS metabolism, reflected by oxygen consumption rate (OCR) levels for basal respiration (Bas. resp.), maximal respiration (Max. resp.), spare capacity (Spare cap.), and ATP production (ATP prod.) and (**C**) anaerobic glycolytic metabolism reflected by extracellular acidification rate (ECAR) levels for glycolysis (Glyco.), glycolytic capacity (Glyco. capacity), and glycolysis reserve (Glyco. reserve) were measured in MiaPaCa-2 cells treated with 10 μM TFP for 24 h. (**D**) ATP production by OXPHOS and anaerobic glycolysis were determined in MiaPaCa-2 cells upon 10 μM TFP-treatment for 24 h. (**E**) Lactate release, (**F**) glucose uptake, and (**G**) glutamine uptake were measured in the extracellular medium after 24 h in culture in TFP and non-treated cells. (**H**) OCR was determined in MiaPaCa-2 cells treated with 10 μM TFP when cells were challenged to UK5099, Etomoxir, and BPTES (inhibitors of glucose oxidation, glutaminase, and carnitine palmitoyl-transferase 1A, respectively). Total RNAs were extracted to monitor the mRNA level of genes involved in the Krebs cycle (**I**) and glycolysis (**J**) using qRT-PCR (fold-change compared with untreated cells). For each treatment, statistical significance is * *p* < 0.05, ** *p* < 0.01, *** *p* < 0.001, and **** *p* < 0.001 compared with untreated cells (Student’s 2-tailed unpaired *t*-Test). Data represent mean ± SEM, *n* = 3 (with technical triplicates).

Once we determined the cell death mechanisms induced by TFP, we were interested in understanding the cellular processes that led to this end. Mechanistically, programmed cell death is induced following a decrease in intracellular availability of ATP [11]. Consequently, we measured the ATP content in cells after TFP-treatment. Unsurprisingly, TFP was able to reduce ATP content in a dose-dependent manner (Figure 2A and Figure S2). Cellular ATP is produced by OXPHOS metabolism, which takes place in the mitochondria, and by anaerobic glycolysis. We carefully studied both sources of ATP production. Regarding the OXPHOS metabolism, we evaluated the O_2_ consumption rate by mitochondria (OCR) by Seahorse technology, in untreated and TFP-treated cells. O_2_ consumption was measured at basal level and after oligomycin, FCCP, and rotenone/antimycin A treatment, to determine the basal respiration, the maximal respiration, the mitochondrial spare capacity, and the ATP production. All the mitochondrial parameters were found strongly decreased upon TFP-treatment (Figure 2B), meaning that OXPHOS metabolism is extremely affected by TFP. Because aerobic ATP production by mitochondria was inefficient in TFP-treated cells, we measured the energy production by anaerobic glycolysis, using the extracellular acidification rate (ECAR) as a read-out. These experiments demonstrated that the anaerobic glycolysis, as well as the glycolytic capacity, which reflects the maximal glycolytic capacity of the cells, was increased in TFP-treated cells. In turn, this higher rate of use of glycolysis strongly reduced the glycolytic reserve in these cells (Figure 2C). Moreover, by measuring the OCR and the proton production rate by cells in the extracellular medium, we can calculate the ATP production by OXPHOS and glycolysis in control and TFP-treated cells.

Our results show that TFP-treatment induces significant changes in the amount of ATP produced by each source. In the control condition, the ATP produced by the OXPHOS metabolism was 92.17 ± 1.95 pmoles/min/1000 cells whereas in TFP-treated cells it dropped to 54.23 ± 3.38 pmoles/min/1000 cells. On the other hand, ATP produced by glycolysis in control cells was 15.01 ± 0.188 pmoles/min/1000 cells, whereas it was raised to 26.51 ± 1.86 pmoles/min/1000 cells in the treated cells, which represents 32.83% of the total ATP produced upon treatment (Figure 2D). Additionally, we validated our previous data by measuring lactate release in the extracellular medium. After 24 h of treatment, lactate release was increased in TFP-treated cells, which is in agreement with a greater glycolytic metabolism (Figure 2E).

With the aim to assess if the drop of intracellular ATP amount may be due to decrease energy substrates availability in the cells, we measured glucose and glutamine uptake. In the TFP-treated cells, we found that whereas glutamine uptake was not modified, the uptake of glucose was strongly augmented in these cells, as showed in Figure 2F,G, indicating that transportation of substrates to inside the cell was not affected by TFP. Then, using the mito-fuel dependency test, we measured the mitochondrial O_2_ consummation depending on the glutamine, fatty acids or glucose as substrates, with the aim to understand if TFP-treatment may induce a decrease of mitochondrial ATP-production depending on the TCA cycle substrate. In control conditions, inhibition of glucose, fatty acids, or glutamine as substrates for ATP production with UK5099, etomoxir, or BPTES, respectively, decreased the O_2_ consummation as expected. On the other hand, O_2_ consumption in TFP-treated cells was lower than in control cells, the inhibition of these substrates did not modify the mitochondrial O_2_ consummation (Figure 2H). These data indicate that mitochondria are not appropriately using these substrates to generate ATP upon treatment. Accordingly, the expression of some enzymes involved in the TCA cycle, such as SDHA, FH, PDHA, and IDH2, was found significantly down-regulated in TFP-treated cells (Figure 2I). Important to note is that several genes involved in glycolysis, such as PDK, GPI, TPI, PGK1, and PGAM1, were concomitantly up-regulated (Figure 2J). Taken together, these results strongly support the hypothesis that under TFP-treatment, the ATP production is switched towards glycolysis instead of OXPHOS. However, glycolysis is less efficient in terms of ATP production than OXPHOS, and the glucose reserve was rapidly consumed. Therefore, we can assume that TFP-treated cells are limited in ATP intracellular availability, which results in programmed cell death.

### 2.3. Trifluoperazine-treatment Induces Mitochondrial and Endoplasmic Coupled Stress

**Figure 3:**
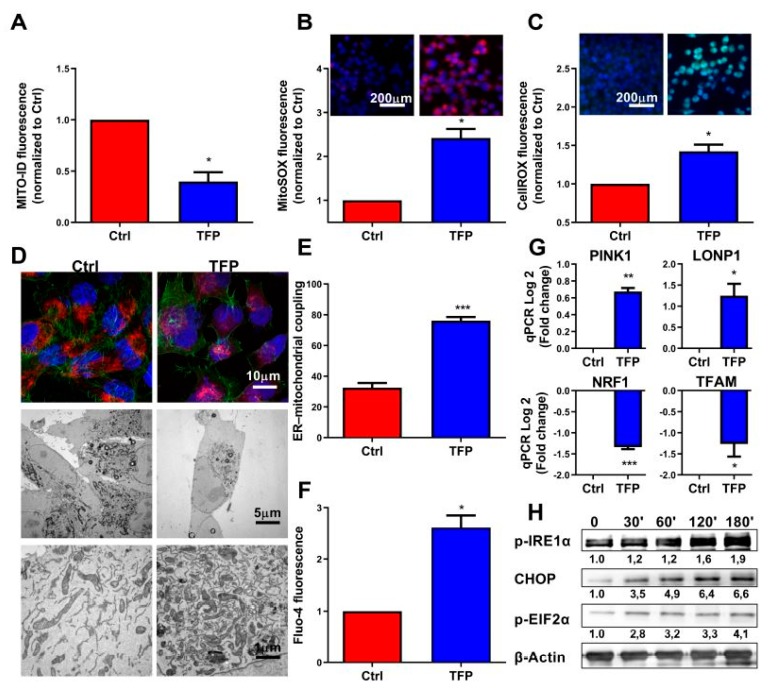
Trifluoperazine promotes mitochondrial and ER coupled stress. (**A**) Flow cytometry analysis of MiaPaCa-2 cells treated with 10 μM TFP for 24 h was done with MITO-ID for analyzing mitochondrial membrane potential. ROS production was detected using MitoSOX Red (**B**) and CellRox Green (**C**) by flow cytometry analysis in TFP and non-treated cells. (**D**) Representative transmission electron microscopy pictures with different magnifications of the MiaPaCa-2 cells after treatment with 10 μM TFP for 24 h are shown. (**E**) Percentage of mitochondria with ER contacts per field (mean of 10 fields) on each condition; not less than 250 mitochondria were counted. (**F**) Flow cytometry analysis with Fluo-4-AM was performed to determine cytosolic calcium concentration. (**G**) Total RNAs were extracted to monitor the mRNA level of genes involved in the mitochondrial dynamic. (**H**) Western blot analysis was performed to evaluated protein levels of expression of Phospho-IRE1α, Phospho-eIF2α, CHOP, and β-actin in TFP-treated cells. For each treatment, statistical significance is * *p* < 0.05, ** *p* < 0.01, *** *p* < 0.001, and **** *p* < 0.001 compared with untreated cells (Student’s 2-tailed unpaired *t*-Test). Data represent mean ± SEM, *n* = 3 (with technical triplicates).

The above results suggested a defect of the OXPHOS mitochondrial activity after TFP-treatment, which is evidenced by the strong deficiency of O_2_ consummation and ATP production. We, therefore, analyzed some features of these mitochondria in TFP-treated cells. Firstly, we measured the mitochondrial membrane potential by using the Mito ID membrane potential kit. The mitochondrial membrane potential is generated by proton pumps (complexes I, III and IV) and plays an important role in the process of energy production during mitochondrial metabolism. However, its persistent drop may be deleterious by inducing cell death. We found a significant decrease in this potential in TFP-treated cells (Figure 3A), which could explain the defect observed in its ATP production. Then, we measured ROS and superoxide production by the mitochondria by using the CellROX and MitoSOX reagents, respectively, by both fluorescent microscopy and flow cytometry. The production of ROS and superoxide anion by mitochondria is important because its accumulation triggers cellular oxidative damage that may induce programmed cell death. Accordingly, intracellular amounts of both ROS and superoxide were significantly augmented in TFP-treated cells as measured by flow cytometry and fluorescence microscopy (Figure 3B,C and Figure S3A). Our results revealed that superoxide and total ROS production were 2.42 ± 0.21 and 1.42 ± 0.09 times greater, respectively, in TFP-treated cells. Then, we analyzed the distribution of the mitochondria into the cells by using the MitoTracker Red reagent in combination with Alexa Fluor 488-phalloidin staining. In TFP-treated cells, the organelles remained localized in a limited region, close to the peri-nuclear region. On the contrary, control cells showed that mitochondria were distributed in the cytoplasm forming an organized network, as expected (Figure 3D). Moreover, we could not observe any evident ultra-structural changes in TFP-treated mitochondria when they were analyzed by transmission electron microscopy, although their previous reported localization was confirmed (Figure 3D). Importantly, we noted that mitochondria of the TFP-treated cells were almost systematically associated with the ER, and the ER was enlarged as it occurs during ER stress, passing from the 32.50% ± 3.044% in non-treated cells to a 76.08% ± 2.37% (Figure 3E). One of the most typical features of the ER stress is the massive Ca^++^ release from the stressed ER to the cytoplasm. We, therefore, measured the cytoplasmic Ca^++^ concentration by TFP-treated cells and found a considerable increase (Figure 3F and Figure S3B). Concomitantly, we found a significant down-regulation of TFAM expression, a gene encoding a key mitochondrial transcription factor functioning in mitochondrial DNA replication and repair, and NRF1, a transcription factor that activates expression of nuclear genes required for respiration and mitochondrial DNA transcription and replication as a possible response mechanism. On the contrary, expression of LONP1, a gene encoding a mitochondrial protease involved in the selective degradation of misfolded, unassembled, or oxidatively damaged polypeptides and PINK1, a serine/threonine protein kinase that it is thought to protect cells from stress-induced mitochondrial dysfunction, were over-activated (Figure 3G). Finally, we performed Western blotting experiments to further confirm if, after TFP-treatment, endoplasmic reticulum homeostasis was disrupted and cells activated the unfolded protein response (UPR). Our results showed an increase of phosphorylation of inositol-requiring enzyme 1 (IRE1α) which is an ER transmembrane sensor that activates the UPR; an upregulation of the phosphorylation of eukaryotic initiation factor 2α (eIF2α), which downregulates protein synthesis in stress condition and an increase of the expression of CHOP, a protein which is induced by ER stress and mediates apoptosis (Figure 3H). Altogether, these results demonstrate a malfunction, as well as an abnormal subcellular distribution of the mitochondria, which may be due to the massive released Ca^++^ from the stressed ER as well as significant oxidative stress and ROS overproduction in TFP-treated cells.

### 2.4. Proteasome System Activity was Activated after Trifluoperazine-treatment

**Figure 4:**
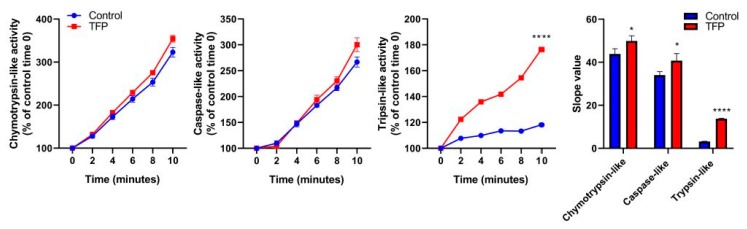
Trifluoperazine increases proteasome proteolytic activity. Kinetics of chymotrypsin-like, caspase-like, and trypsin-like activities of the proteasome in MiaPaCa-2 cells after 48 h of TFP-treatment with 10 μM were measured. After the addition of the fluorogenic substrates, fluorescence was recorded over time at 37 °C for 2 min time intervals. The slope values of the curves were calculated. For each treatment, statistical significance is * *p* < 0.05, ** *p* < 0.01, *** *p* < 0.001 and **** *p* < 0.001 compared with untreated cells (Student’s 2-tailed unpaired *t*-Test). Data represent mean ± SEM, *n* = 3 (with technical triplicates).

ER stress response is accompanied by overexpression and post-translational modification of ER stress-associated proteins [12] as well as the activation of the ubiquitin-proteasome system (UPS). The UPS is a major intracellular protein degradation complex that induces the clearance of misfolded ubiquitinated proteins by the proteasome. The proteasome is a protein complex containing a central part composed of four stacked rings. Each ring is composed of seven individual proteins, denominated α, the two external, and β, the two inners. The proteolytic chamber of the proteasome is formed by the two β-rings. They harbor the three catalytic subunits, named β1, β2, and β5, which show caspase-like, trypsin-like, and chymotrypsin-like activities, respectively. As we previously observed that TFP-treated cells underwent ER-stress, which normally led to an accumulation of misfolded proteins, we therefore evaluated if the activity of the proteasome system remains active. We found that the activity of the proteasome in its three catalytic functions was activated. The chymotrypsin-like and the caspase-like activities of the proteasomes had a smaller increase of activity, whereas the trypsin-like activity was greatly increased in TFP-treated cells compared to the untreated, which curve slope moved from 3.18 ± 0.05 to 13.79 ± 0.09 (Figure 4). This result indicates that after TFP-treatment, proteasome activity is still active to offset ER-stress.

### 2.5. Proteasome Inhibition Increases Sensitivity to Trifluoperazine of Primary Pancreatic Cancer Cells

**Figure 5:**
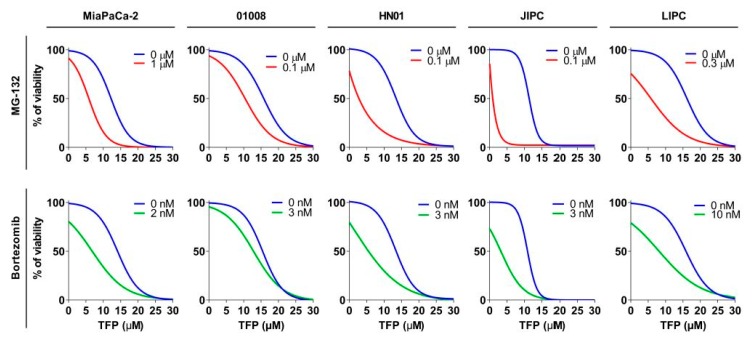
Trifluoperazine treatment in combination with proteasome activity inhibitors has a synergic anti-cancer effect. Chemograms of MiaPaCa-2 and 4 PDX-derived cell lines were performed at increasing concentrations of TFP in combination with fixed concentrations of MG-132 or bortezomib after 24 h of treatment. Cell viability is indicated in % to the control (vehicle-treated).

Since TFP-treated cells activate the UPS to overcome ER-stress as a salvage pathway, we, therefore, speculated that decreasing the proteasome activity in TFP-treated cells could be a useful approach. We used different proteasome inhibitors: bortezomib, which is an inhibitor of subunit β5 (with chymotrypsin-like activity), or MG-132, which targets the three subunits. For this experiment, we used an MiaPaCa-2 cell line and 4 PDAC-derived primary cells-01008, HN01, JIPC, and LIPC. The viability experiments were carried out, incubating the cells with increasing concentration of TFP (from 0 to 30 µM), in the presence or not of MG-132 or bortezomib at different concentration depending on the cell sensibility. When TFP was used as a unique treatment, the IC_50_ ranged from 10.46 to 15.07 µM depending on the cell, but after combination with MG-132, this range decreased until 0.87 from 8.05 µM and in the case of bortezomib until 2.02 from 8.65 µM, as showed in Figure 5 and Table 1. Hence, this data supports the hypothesis stated above by showing that TFP-treatment is not only efficient but, in addition, its anticancer activity can be enhanced by proteasome inactivation.

## 3. Discussion

PDAC is an aggressive disease with a very low rate of survival (7% in 5 years) [13]. For this reason, new therapeutic approaches are needed to improve the life expectancy of the patients. Designing, synthesizing, and testing new compounds is a possibility; however, the improvement of them is extremely costly and time-consuming for a disease with no curative chemotherapy. For this reason, we have focused our attention on the TFP drug. Previously, we have rationally repurposed TFP [8], an antipsychotic drug, for treating PDAC, and others have demonstrated its anticancer activity in other cancers [4,14,15]. Nevertheless, the mechanisms of action by which TFP induces cell death are poorly understood.

In this work, we disclose that TFP-treatment is able to induce cell death by apoptosis and necroptosis in MiaPaCa-2 cells. Moreover, cell viability was rescued by using cell death inhibitors, Z-VAD-FMK and Necrostatin-1, for apoptosis and necroptosis, respectively. Cancer resistance is common in chemotherapy but frequently leads to therapeutic failure and acquired resistance to apoptosis. Thus, the induction of several cell death pathways by the TFP-treatment represents a therapeutic advantage. Apoptosis and necroptosis are cell death pathways in which ATP plays an important role and determines cell fate. Accordingly, we found a strong ATP level depletion in TFP-treated cells. This dramatic drop of ATP level was the consequence of a disruption of the OXPHOS metabolism, accompanied by a compensatory increase of anaerobic glycolysis. However, this transitory energetic shift was unable to compensate for the loss of the mitochondrial function. Furthermore, we disclose that the mitochondrial failure is due to a great loss of the mitochondrial membrane potential, a strong increase in ROS production, and concomitant relocalization of mitochondria to the vicinity of the endoplasmic reticulum. Mitochondria and energy metabolism disruption play an important role in cell death. This is a hot topic, and nowadays, several therapeutic approaches are exploiting and targeting it, as is the case of metformin, currently under clinical trials [16,17]. In this regard, TFP-treatment demonstrates a great anticancer effect because it is able to target mitochondria and induce metabolic-driven cell death.

Remarkably, we observe an increased interaction between mitochondria and ER. These organelles form the structural and functional networks called MAMs (mitochondria-associated ER membranes). The equilibrium in the interchange of proteins, lipid, ROS, or ions (Ca^++^) between the two cellular structures is essential for cellular survival [18]. It is well known that cancer cells are subjected to an increased cellular stress, that counter by enhancing the antioxidant cellular machinery, like GSH-related enzymes [19], or by the overexpression of ion channel as IP3R/Ca^++^ channels [20], among others. However, sustained overproduction of cellular ROS species and superoxide anion (the last one in the mitochondria) and maintained increase of Ca^++^ levels in the cytosol triggers a vicious circle of mitochondrial and ER-coupled stress. In this regard, we observed an increase in ROS production and the release of Ca^++^ to the cytoplasm after TFP treatment. In addition, phosphorylation of IRE1α and eIF2α and overexpression of CHOP proteins, classical markers of ER-stress, were found to increase in treated cells. Thus, the TFP-treated cells suffered from a mitochondrial–ER coupled stress that they were not able to overcome, inducing in turn, programmed cell death.

In order to bypass ER-stress, cells activate the UPR. This mechanism degrades the unfolded or misfolded proteins and thereby protects the cell from programmed death. We took advantage of this information and used MG-132 or bortezomib, the last one as an approved drug for cancer treatment [21], to block the activity of the proteasome. With the co-treatment, we inhibited this mechanism of protection that was enhanced by the TFP treatment. Therefore, the proteasome inhibitors increased the accumulation of unfolded and misfolded proteins, being able to improve the efficacy of TFP for treating pancreatic cancer. Remarkably, by combining TFP and the proteasome inhibitors, we were able to increase up to 10 times the killing potential of the TFP in some PDAC-derived primary cells. In our previous studies in vivo [8], we observed the anti-tumor activity of TFP at 5 to 10 mg/Kg, as a single treatment. Zhang et al. [10] reported that TFP can be used at 1 mg/Kg without any neurological side-effect. In agreement with our in vitro experiment, where the doses were reduced 10 times, TFP can be used in vivo at 1 mg/Kg in combination with proteasome inhibitors retaining its anti-tumor effect. More interestingly, tumor cells in PDAC are extremely challenged to hypoxic conditions and TFP showed strong anti-tumoral effect also in hypoxic conditions. In this line, it has been shown that bortezomib inhibits tumor adaptation to hypoxia by repressing HIF-1α expression and sensitizes cancer cells in hypoxia [22], supporting the combination of TFP and proteasome inhibitors for treating hypoxic tumors such as PDAC. These results are clinically relevant because on one hand, bortezomib is an approved proteasome inhibitor already used in the clinic, and, on the other hand, since the main problem of using TFP as an anticancer drug in patients is its neurological side-effects, the combination of both drugs could solve this problem by using smaller doses. In this regard, different clinical trials are studying TFP administration in patients, which will lead to a better understanding about the doses that can be administered without side-effects, as well as the treatment of pancreatic adenocarcinoma with proteasome inhibitors. Actually, further clinical trials in progress study the combination of proteasome inhibitors with multiple anticancer drugs. As TFP has shown strong anti-cancer activity and synergism with different proteasome inhibitors, combinatory therapy could be a promising strategy for pancreatic adenocarcinoma.

In conclusion, we have identified that TFP-treatment induces cell death by apoptosis and necroptosis due to coupled endoplasmic reticulum and mitochondrial stress. More importantly, degradation by UPS behaves as a salvage pathway for these phenomena. This finding led us to efficiently test the use of proteasome inhibitors in combination with TFP (MG-132 and bortezomib). In this regard, we found an important synergism consequently increasing the anticancer effect of the TFP. Thus, the current study not only uncovers the molecular mechanisms that are triggered upon TFP-treatment but also through its use with bortezomib, both FDA-approved drugs available for pancreatic cancer treatment. Repurposing them as combinatory therapy may lead to developing new treatments for pancreatic adenocarcinoma.

## 4. Materials and Methods

### 4.1. Cell Culture

MiaPaCa-2 cells were purchased in the American Type Culture Collection and cultured in Dulbecco’s modified Eagle’s medium (DMEM) (Gibco, Life Technologies, Carlsbad, CA, USA) supplemented with 10% fetal bovine serum (Lonza, Basel, Switzerland) and incubated in a humidified atmosphere containing 5% CO_2_ at 37 °C. MiaPaCa-2 cells in all experiments were below 20 passages. PDAC primary cell cultures were obtained from xenografts as previously described [23]. Tissues were split into several small pieces and processed in a biosafety chamber. After a fine mincing, they were treated with collagenase type V (Sigma, Madrid, Spain) and trypsin/EDTA (Gibco, Life Technologies) and suspended in DMEM with 1% *w/w* penicillin/streptomycin (Gibco, Life Technologies) and 10% fetal bovine serum. After centrifugation, cells were re-suspended in serum-free ductal media (SFDM) without antibiotics and incubated at 37 °C in a 5% CO_2_ incubator. To avoid any supplementary stress, all media were preheated at 37 °C before rinsing or changing media. All the PDAC primary cell lines in all experiments were below 10 passages.

### 4.2. LDH Assay, ATP Production, and Caspase-3/7 Activity Assay

Cells were seeded at a density of 10,000 cells per well in 96-well plates. Cells were allowed to attach overnight and treated the next day for 24 h and further incubated for 24 h for the assay. After the incubation, ATP production, LDH release, and caspase-3/7 activity were measured using CellTiter-Glo (#G7571, Promega, Madrid, Spain), CytoTox-ONE (#G7890, Promega), and Caspase-Glo 3/7 assay (#G8091, Promega). The normalization of the data was done by the number of cells.

### 4.3. qRT-PCR

Total RNA was extracted from cells using Trizol reagent (Invitrogen, Carlsbad, CA, USA) and cDNA was obtained by reverse-transcribed using Go Script (Promega), according to the manufacturer’s instructions. Real-time quantitative PCR (qRT-PCR) was performed in a Stratagene MXPro-MX3005P using Promega reagents. Primer sequences are available upon request.

### 4.4. Western Blot Analysis

Proteins were resolved by SDS-PAGE, and transferred to nitrocellulose membranes for 1 h. Then, membranes were blocked 1 h at room temperature with TBS (tris-buffered saline), 5% BSA, and blotted overnight in TBS 5% BSA containing primary antibodies at 1:500 overnight with corresponding antibodies at 4 °C. Subsequently, the blot was washed and incubated with HPR-conjugated secondary antibody (Boster, Pleasanton CA, USA) for 1 h at room temperature at 1:5000 before being revealed with ECL (enhanced chemo-luminescence). The acquisition was performed with a Fusion FX7 imager (Vilber-Lourmat, Sud Torcy, France). The following primary antibodies were used: rabbit polyclonal anti-PARP (#9542, Cell Signalling, Danvers MA, USA), mouse polyclonal anti-CHOP (#2895, Cell Signalling), rabbit polyclonal anti-Phospho-IRE1α (#PA1-16927, Thermofisher Scientific), rabbit polyclonal anti-Phospho-eIF2α (#3398, Cell Signalling), and mouse monoclonal β-actin (#A5316, Sigma).

### 4.5. Extracellular Flux Assay

Measurements were performed with a Seahorse Bioscience XF-24 metabolic flux analyzer. This system allowed measurement of cellular oxygen consumption rate (OCR, in pmoles/minute) extracellular acidification rate (ECAR in mpH/minute), and PPR (proton production rate). 30,000 cells/well were plated onto Seahorse 24-well plates and treated 24 h before the assay. OCR was measured using an XF cell mito stress test kit under basal conditions, and in response to oligomycin 1 μM and FCCP 0.25 in MiaPaCa-2. After oligomycin injection, we calculated the OCR for the ATP production, and after FCCP treatment, we were able to assess the maximal OCR capacity and the spare respiratory capacity. At the end of the experiment, the mitochondrial respiration was stopped by adding the electron transport chain inhibitors rotenone and antimycin A (0.5 μM each). We measured ECAR by using the Seahorse XF glycolysis stress test kit. We calculated the glycolysis values as the difference between the values of ECAR upon glucose injection at 10 mM and basal ECAR. We measured the glycolytic capacity as the difference between ECAR upon the injection of oligomycin (1 μM), and the basal ECAR. In addition, we assessed the glycolytic reserve as the difference in ECAR between glucose and oligomycin treatments. Finally, glycolysis was inhibited by adding 2-Deoxyglucose at 100 mM. OXPHOS and glycolysis contribution to ATP production was calculated with OCR and PPR data, as previously described [24]. In order to determine the rate of oxidation of each fuel, the Seahorse XF mito fuel flex test was used. Inhibitors of glutaminase (BPTES 3 μM), carnitine palmitoyl-transferase 1A (Etomoxir 4 μM), and glucose oxidation (UK5099 2 μM) were used. Levels of OCR, PPR, and ECAR were normalized to the cell number.

### 4.6. Lactate Release, Glucose and Glutamine Uptake

A YSI 2900 Biochemistry Analyzer (YSI Life Sciences, Yellow Springs, OH, USA) was used to determine the levels of glucose, glutamine, and lactic acid in the culture media, following the manufacturer’s protocol. Differences in lactate production were calculated by subtracting the concentration of lactate present in the control medium from the lactate present in the media after incubation. Glucose and glutamine uptake was calculated by subtracting the glucose levels of the experimental media from the glucose level of the control medium.

### 4.7. Annexin V/PI Staining

After 24 h of incubations with the stimuli, cells were collected. Briefly, cells were washed with PBS and then detached with accutase (Gibco, Life Technologies) and resuspended in 100 μL of 1× annexin V-binding buffer. The reagent Pacific-Blue Annexin V (5 μL, BioLegend, San Diego, CA, USA) was added to the cell suspension for 15 min and incubated at room temperature. Then 400 μL of 1× annexin V-binding buffer was added to each sample. Propidium iodide (5 μL, Miltenyi Biotec, Bergisch, Germany) was added to the suspension before the cytometry analysis. Ten thousand cell per sample were collected in a MACSQuant-VYB (Miltenyi Biotec, Surrey, UK). Data analysis was carried out by using the FlowJo software.

### 4.8. ROS Measurements

CellRox Green and MitoSOX (Molecular Probes, Carlsbad, CA, USA) were added to a final concentration of 5 μM during 30 and 10 min, respectively. Then, cells were washed with warm PBS and detached with accutase and resuspended in HBSS (Gibco, Life Technologies) for flow cytometry. Cells were fixed with 4% paraformaldehyde for 10 min at room temperature, then washed with PBS and mounted with Prolong Gold antifade reagent with DAPI (Invitrogen, Carlsbad, CA, USA). For the cytometry experiments, 10,000 cells per sample were analyzed in a MACSQuant-VYB. Data analysis was performed by using FlowJo software. Image acquisition was carried out on a microscope Nikon Eclipse 90i fluorescence microscope.

### 4.9. Mitochondrial Membrane Potential

Measurement of mitochondrial membrane potential was performed by using a MITO-ID Membrane potential detection kit (ENZ-51018) following the manufacturer’s protocol (Enzo Life Sciences, Farmingdale, NY, USA). After incubation, cells were collected, washed with PBS. Then samples were preincubated in 500 μL of the assay solution containing 5 μL of MITO-ID MP detection reagent for 15 min. Finally, 10,000 events per sample were collected in a MACSQuant-VYB and analyzed using FlowJo software.

### 4.10. Cytosolic Calcium Concentration

Fluo-4 AM (Molecular Probes) was used to pre-incubate the cells for 30 min at 37 °C. Then, cells were washed with PBS and resuspended in HBSS medium before cytometry analysis. Ten thousand events per sample were collected in a MACSQuant-VYB and analyzed by using FlowJo software.

### 4.11. Mitochondrial Network

Mitochondrial network localization was studied after incubation of cells with a MitoTracker DeepRed FM (200 nM, Molecular Probes) at 37 °C for 30 min. After that, cells were washed twice with PBS solution and fixed with 4% paraformaldehyde for 10 min. Subsequently, cells were permeabilized with 0.1% Triton X-100 and blocked with 1% BSA., Then, staining with Alexa Fluor 488 phalloidin was performed following the manufacturer’s instructions. Finally, samples were mounted using Prolong Gold antifade reagent with DAPI. Confocal images of mounted samples were acquired using an inverted microscope, using a 63× lens and equipped with LSM 880 with an Airyscan detector controlled by Zeiss Zen Black software.

### 4.12. Electron Microscopy

Cells were treated following the NCMIR protocol for SBF-SEM53. Seventy nm-ultrathin sections were cut using a Leica UCT Ultramicrotome (Leica, Vienna, Austria) and transferred to formvar-coated slot grids. Samples were observed in an FEI Tecnai G2 at 200 KeV and image acquisition was performed on a Veleta camera (Olympus, Tokyo, Japan).

### 4.13. Chemograms and Viability Assays

Five thousand cells per well were plated in 96-well plates in SFDM or DMEM medium. The media was supplemented with increasing concentrations of TFP, MG-132, or bortezomib with a Tecan D300e Digital Dispenser during 24 h. Cells were then incubated for an additional 72 h period. Cell viability was estimated after the addition of PrestoBlue reagent (Life Technologies, Carlsbad, CA, USA) for 3 h, following the supplier protocol. Cell viability was normalized with respect to untreated cell rates.

### 4.14. Proteasome Activity Assay

MiaPaCa-2 cells were seeded in a 10 cm dish and treated when they reached 70% confluence. Briefly, cells were lysed and sonicated and the supernatant was collected. Proteasome activities were measured using a proteasome activity fluorometric assay kit II (#J4120, UBPBio, Aurora, CO, USA), according to the manufacturer’s instructions. Protein extract was mixed with 50 μM of fluorogenic substrates (UBPBio) in 1x proteasome assay buffer in a total volume of 100 μL. Samples with assay buffer only (no substrate) served as the control for autofluorescence. Sample fluorescence was quantified every 2 min for 10 min using a 360/460 nm filter by a TECAN infinite 96-plate reader at 37 °C.

### 4.15. Statistics

Statistical analyses were performed using the unpaired 2-tailed Student *t*-Test and non-normal distribution or 1-way ANOVA with Tukey’s post hoc test when appropriate. A *p*-Value less than or equal to 0.05 was considered significant. All values are expressed as mean ± SEM. Data are representative of at least 3 independent experiments with technical triplicates completed.

## 5. Conclusions

In conclusion, we identified that TFP-treatment induces cell death by apoptosis and necroptosis due to coupled endoplasmic reticulum and mitochondrial stress. More importantly, degradation by UPS behaves as a salvage pathway for these phenomena. This finding led us to efficiently test the use of proteasome inhibitors in combination with TFP (MG-132 and bortezomib). In this regard, we found an important synergism and, consequently, increasing anticancer effect of TFP. Thus, the current study not only uncovers the molecular mechanisms that are triggered upon TFP-treatment but also through its use with bortezomib, both FDA-approved drugs available for pancreatic cancer treatment. Repurposing them as combinatory therapy may lead to developing new treatments for pancreatic adenocarcinoma.

## Figures and Tables

**Table 1:** IC_50_ and area under the curve (AUC) of PDAC cell lines upon 24 h of treatment with TFP alone or in combination with MG-132 or bortezomib.

Cell Line	IC50 (µM)			AUC (a.u.)		
	TFP	TFP + MG-132	TFP + bortezomib	TFP	TFP + MG-132	TFP + bortezomib
MiaPaCa-2	13.24 ± 0.21	5.46 ± 0.17 ****	6.81 ± 0.20 ****	1372.67 ± 101.18	618.13 ± 49.26 ****	939.37 ± 19.02 ****
1008	10.46 ± 0.48	8.05 ± 0.10 **	8,65 ± 0.11 *	1507.67 ± 49.97	1260.00 ± 10.78 **	1140.67 ± 15.16 **
HN01	14.78 ± 0.044	2.47 ± 0.36 ****	3.16 ± 0.29 ****	1345.67 ± 15.026	525.7 ± 9.05 ****	709.37 ± 24.60 ****
JIPC	11.45 ± 0.067	0.86 ± 0,01 ****	2.02 ± 0.44 ****	1218 ± 14.98	242.5 ± 1.84 ****	425.43 ± 34.75 ****
LIPC	15.07 ± 8.07	5.81 ± 0.08 ****	8.15 ± 0.09 ****	1559 ± 28.583	754.1 ± 9.15 ****	917 ± 16.49 ****

For each treatment, statistical significance is * *p* < 0.05, ** *p* < 0.01, *** *p* < 0.001, and **** *p* < 0.001 compared with untreated cells (Student’s 2-tailed unpaired *t* Test). Data represent mean ± SEM, *n* = 3 (with technical triplicates).

## Supplementary Materials

The following are available online at https://www.mdpi.com/2072-6694/11/12/1869/s1, Figure S1: Trifluoperazine induces cell death via apoptosis and necroptosis in PDAC cells, Figure S2: Trifluoperazine decreases ATP production in PDACcells, Figure S3: Trifluoperazine promotes mitochondrial and ER coupled stress.
