# Supplementary material for: Dissecting the Anticancer Mechanism of Trifluoperazine on Pancreatic Ductal Adenocarcinoma
Source: Cancers (Basel). 2019 Nov 26;11(12):1869. doi: 10.3390/cancers11121869 (PMC6966621; doi:10.3390/cancers11121869)
Supplement: Supplementary file 1 [file cancers-11-01869-s001.pdf]

Supplementary Materials

Dissecting the Anticancer Mechanism of Trifluoperazine on Pancreatic Ductal Adenocarcinoma

Can Huang, Wenjun Lan, Nicolas Fraunhoffer, Analía Meilerman, Juan Iovanna and Patricia Santofimia-Castaño

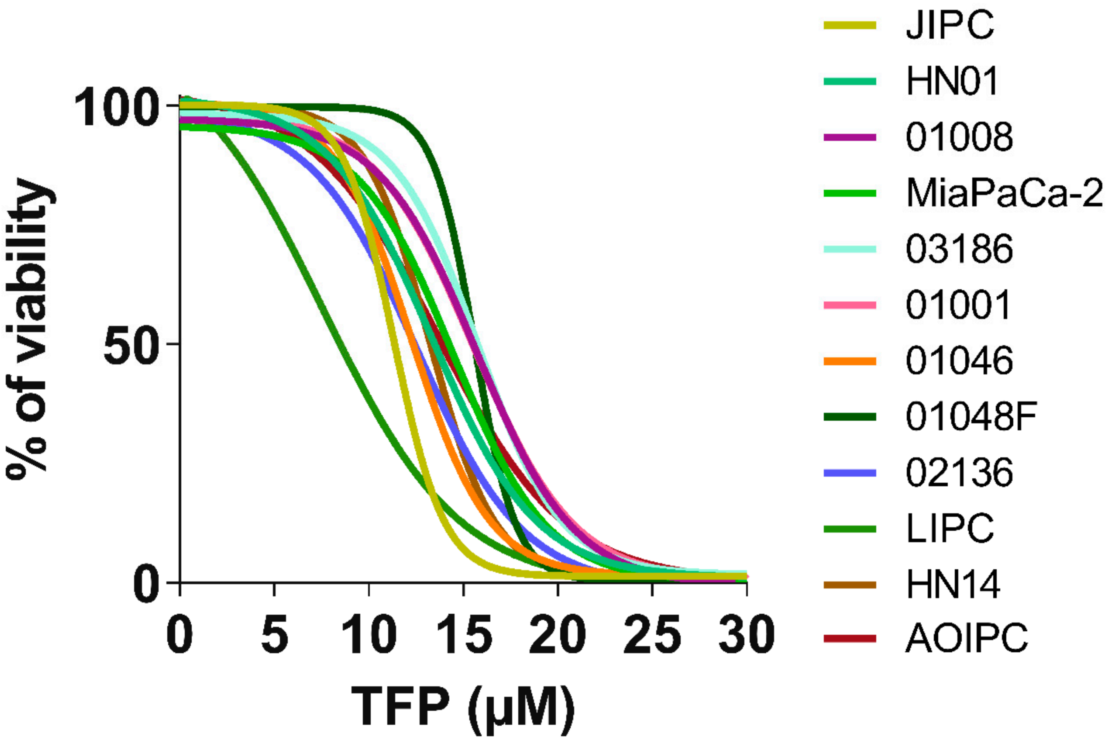

Figure S1. Trifluoperazine induces cell death via apoptosis and necroptosis in PDAC cells. Viability of MiaPaCa-2 cells and 11 PDX-derived cell lines upon a 24-h treatment with TFP at increasing concentrations of trifluoperazine was measured.

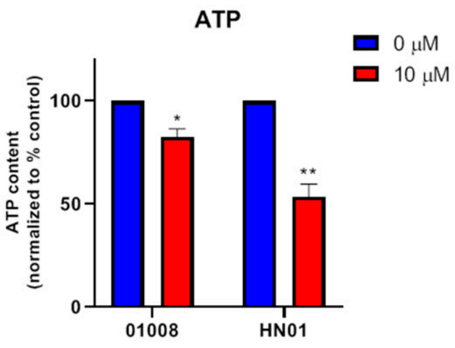

Figure S2. Trifluoperazine decreases ATP production in PDACcells. Cells were incubated with TFP at 10 µM, and ATP content was measured after 24 h of treatment. For each treatment, statistical sig-

nificance is * $p < 0.05$, ** $p < 0.01$, compared with untreated cells (Student's 2-tailed unpaired *t*-Test). Data represent mean ± SEM, $n = 3$ (with technical triplicates).

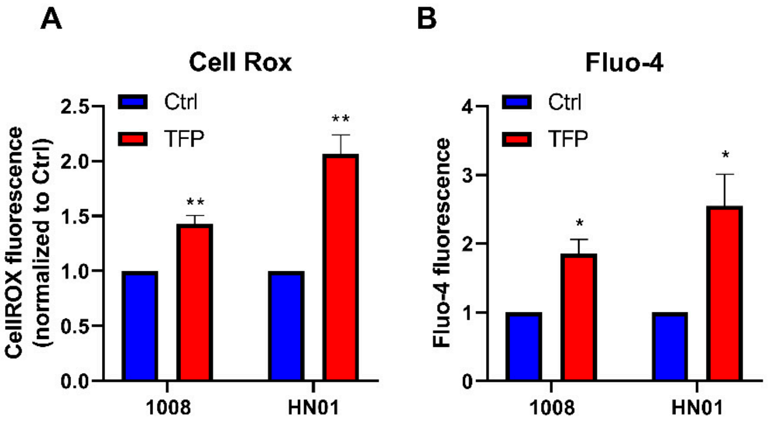

Figure S3. Trifluoperazine promotes mitochondrial and ER coupled stress (**A**) ROS production detected using MitoSOX Red by flow cytometry analysis in TFP and non-treated cells. (**B**) Flow cytometry analysis with Fluo-4-AM performed to determine cytosolic calcium concentration. For each treatment, statistical significance is * $p < 0.05$, ** $p < 0.01$, compared with untreated cells (Student's 2-tailed unpaired *t*-Test). Data represent mean ± SEM, $n = 3$ (with technical triplicates).

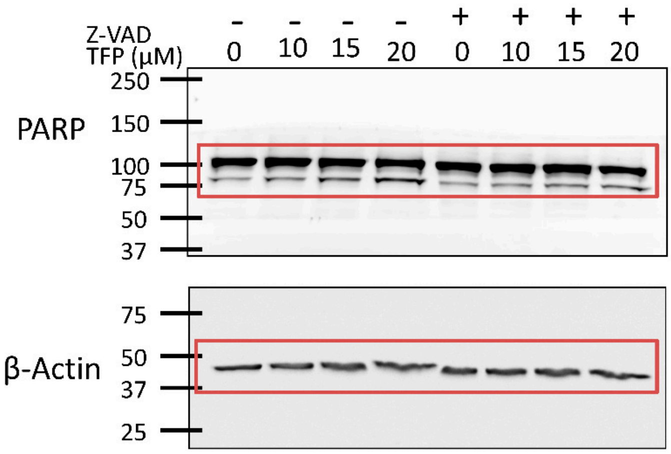

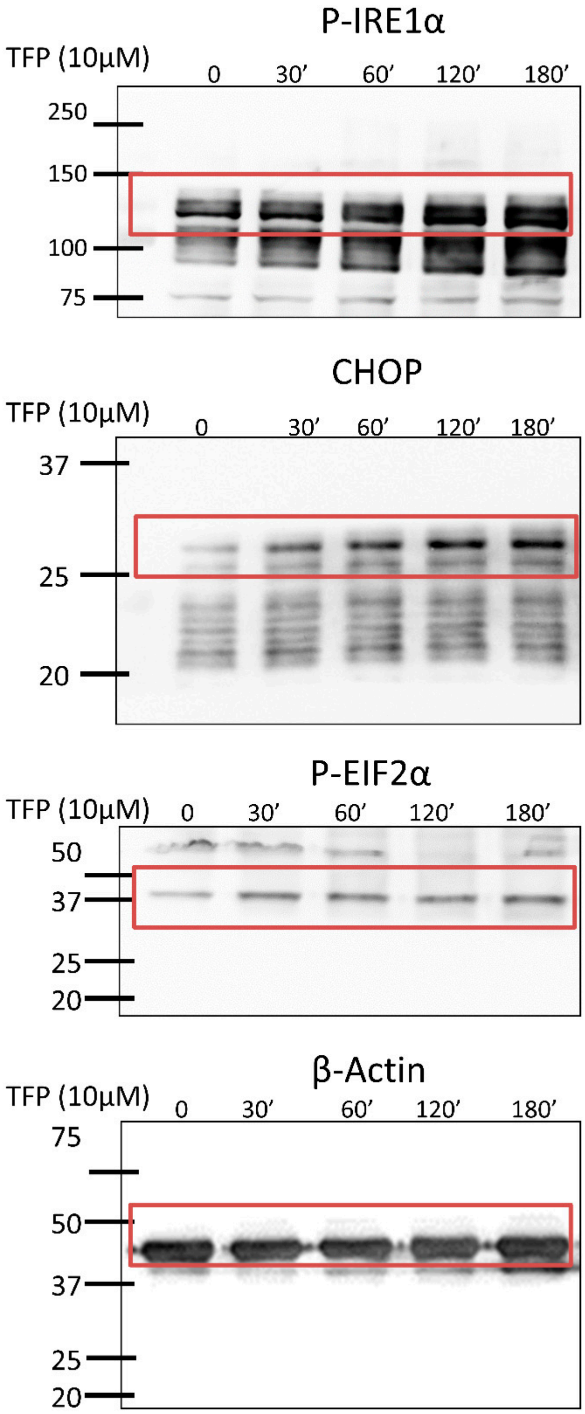

© 2019 by the authors. Licensee MDPI, Basel, Switzerland. This article is an open access article distributed under the terms and conditions of the Creative Commons Attribution (CC BY) license (http://creativecommons.org/licenses/by/4.0/).
